# Complement C3: an emerging risk factor in cardiometabolic disease

**DOI:** 10.1007/s00125-012-2462-z

**Published:** 2012-01-27

**Authors:** E. Hertle, M. M. J. van Greevenbroek, C. D. A. Stehouwer

**Affiliations:** 1 Laboratory for Metabolism and Vascular Medicine, Department of Internal Medicine (UNS 50 / box 14), Maastricht University Medical Centre, PO Box 616, 6200 MD Maastricht, the Netherlands; 2 CARIM School for Cardiovascular Diseases, Maastricht University, Maastricht, the Netherlands

**Keywords:** Atherothrombosis, Cardiometabolic disease, Complement activation, Complement C3, Fibrinolysis, Hypofibrinolysis, Macrovascular disease, Microvascular disease

## Abstract

C3 is the central component of the complement system and activation of C3 via any of the three major activation pathways—the classical, the lectin and the alternative pathways—results in initiation of the terminal complement pathway and release of the anaphylatoxin C3a. Both terminal pathway activation and signalling of C3a and its inactivation product C3a-desarg via the C3a receptor and C5a-like receptor 2, respectively, can induce inflammatory, immunomodulatory and metabolic responses. C3 has been implicated in metabolic disorders, notably adiposity, dyslipidaemia, insulin resistance, liver dysfunction and diabetes, and C3 is increasingly recognised as a cardiometabolic risk factor. C3 may play a role in the macrovascular, as well as microvascular, complications of diabetes. Moreover, C3 may interact with the coagulation system and as such also contribute to a procoagulant, hypofibrinolytic and, ultimately, prothrombotic state. Recent data suggest a diabetes-dependent incorporation of C3 into fibrin clots, with concomitant effects on clot characteristics. Taken together, epidemiological and experimental evidence concordantly point to a role of complement C3 in metabolic, atherosclerotic/atherothrombotic and microangiopathic processes and further research should be directed towards the elucidation of complement function and activation in cardiometabolic disorders.

The complement system, a complex protein network initially identified as part of the innate immune system, is increasingly recognised as an essential regulator of cell and tissue homeostasis. It consists of soluble and membrane-bound proteins functioning in cascades of stepwise protease activation; effector functions include the release of anaphylatoxins and formation of terminal complement complexes (TCCs). Anaphylatoxins operate by binding to their receptors on a variety of immune and non-immune cells, where they exert proinflammatory, immunomodulatory and metabolic effects. TCCs are assembled upon membranes, where they exert stimulatory effects on cell cycle and cell metabolism (sublytic TCCs) or promote cytolysis when present in higher amounts (lytic TCCs, also referred to as membrane-attack complexes [MACs]).

C3 lies at the heart of the complement network, as all three major activation pathways may result in cleavage of C3 and initiation of the downstream terminal pathway. Systemic levels of C3 may reflect the potential for complement activation. Upon activation of C3, C3a and C3b are generated. Once formed, the anaphylatoxin C3a is rapidly desarginated by a carboxypeptidase, generating C3a-desarg. Although this was previously thought to be an inactivation process, C3a-desarg has been recognised as a lipogenic hormone and is now also known as acylation-stimulating protein (ASP) [1]. C3b is instrumental in the activation of the terminal pathway of complement activation, which leads to formation of TCCs/MACs (Fig. 1).

**Fig. 1:**
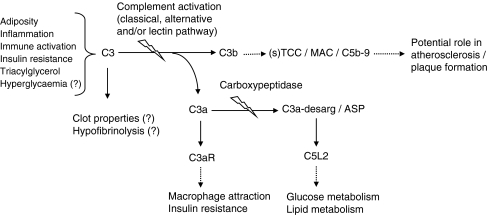
Adiposity, inflammation, immune activation, insulin resistance, hypertriglyceridaemia and, potentially, hyperglycaemia may lead to increased systemic levels of C3. C3a and C3b are generated upon activation of C3. C3b is part of the multi-step complement activation cascade that eventually leads to the formation of soluble (s) TCCs/MACs. The anaphylatoxin C3a is rapidly degraded into its desarginated form, which is also known as ASP. Both C3a and C3a-desarg/ASP can, via binding to their respective receptors (C3aR and C5L2), exert relevant effects with respect to diabetes and CVD. The solid arrows denote direct effects, the dotted arrows denote more distal effects and the lightening bolts indicate activation

Recent data suggest that C3 plays a role in metabolic disorders. For example, a high systemic concentration of C3 was shown to be independently associated with the incidence of type 2 diabetes, at least in men [2]. Additionally, systemic C3 levels have been associated with several diabetes risk factors, including adiposity (waist, BMI), serum triacylglycerol and insulin resistance (as reviewed in [3]). Plasma C3 levels are also higher in non-alcoholic fatty liver disease (NAFLD) [4], a common comorbidity associated with obesity and in type 2 diabetes, and possibly also in type 1 diabetes; in agreement with this observation, alanine aminotransferase—a marker of liver dysfunction—was also associated with plasma levels of C3 [5]. The link between systemic C3 and adiposity is substantiated by the observations that adipose tissue secretes C3, that weight gain is associated with an increase in C3 and that C3 decreases upon weight loss [3]. Further evidence supporting a potential role for C3 in (the development of) diabetes and associated metabolic disorders is the strong association of C3, which is an acute phase reactant, with inflammatory markers [6]. In addition, complement activation can promote systemic inflammation [7].

At least two signalling pathways have been identified that may contribute to the association of C3 activation with insulin resistance, adipose tissue function and lipid metabolism. First, expression of the C3a receptor (C3aR) is particularly high in white adipose tissue and is strongly upregulated after a high-fat diet [8]. Interruption of the C3–C3a–C3aR axis in a *C3aR* ^−/−^ mouse model prevented diet-induced insulin resistance [8]. Second, interruption of the C3–(C3a)–C3a-desarg/ASP–C5a-like receptor 2 (C5L2) pathway in a *C5L2* ^–/–^ mouse model had multiple metabolic effects, including reduced triacylglycerol synthesis in adipose tissue and delayed triacylglycerol and glucose clearance [9]. Reduced signalling of ASP via C5L2 (ASP resistance) may contribute to altered adipose tissue metabolism in obesity/insulin resistance phenotypes in humans [10]. Overall, substantial evidence points towards an active role of C3, at least partly via C3 activation, in diabetes-related metabolic diseases. However, the causal relationships and order of the events that occur in the vicious cycles of adiposity, insulin resistance and inflammation, remain to be unravelled.

C3 may also play a role in the cardiovascular complications of diabetes and related metabolic diseases. C3 has repeatedly been shown to be associated with different manifestations of prevalent and incident cardiovascular disease (CVD), often—but not always—independently of classical risk factors [3]. The observation that the associations with CVD risk factors and disease manifestations are not uniform suggests that there may be distinct properties of C3 contributing to the development of CVD. Mechanistically, the involvement of C3 in the atherosclerotic process is illustrated by the presence of C3 and its activation products in atherosclerotic plaques. A causal role was deduced from animal studies showing that deposition of lipids and complement in the intima precedes the infiltration of inflammatory cells and foam cell development [11].

The role of complement activation in diabetic vascular complications may not be confined to macrovascular disease. The complement system may also contribute to compromised microvascular function, and activation of complement, including C3, may thus contribute to the diabetic nephropathy, retinopathy and neuropathy. Its role in nephropathy is supported by the presence of activated C3 in glomeruli and glomerular capillaries of animal models of type 1 and type 2 diabetes [12, 13]. Furthermore, C3 activation may aggravate existing kidney disease since non-selective filtration of complement proteins may result in intratubular complement activation and tubular damage [14, 15]. Support for a role of C3 in retinopathy and neuropathy is provided by data indicating that activated C3 and TCC/MAC (representing terminal pathway activation) are present in the retinal vessels [16] and choriocapillaries [17] of patients with diabetic retinopathy and in the walls of endoneurial microvessels of patients with diabetic neuropathy [18].

Activation of the complement system may contribute to diabetic vasculopathy via interactions with the coagulation system. Based on the early observation that the complement system is activated during blood clotting, multiple interactions between the complement and the coagulation/fibrinolysis cascades have been revealed (as reviewed in [19]). Several enzymes of the coagulation/fibrinolysis cascade can activate C3 and, conversely, several components of the complement system appear to be involved in the activation of thrombin and the modulation of platelet aggregation. These observations position the complement system as part of a complex protease network that is characterised by substantial crosstalk between complement and the coagulation/fibrinolysis system. This considerable interplay might represent another cluster of proteins in which complement participates in macro- and microvascular disease risk by conferring procoagulant, hypofibrinolytic and ultimately prothrombotic properties.

In this issue of *Diabetologia*, Hess and colleagues present novel aspects of the role of C3 in diabetes with regard to CVD risk [20]. They propose a mechanism in which C3 participates in a hypofibrinolytic, and thus prothrombotic, state. It had previously been recognised that C3 is incorporated into fibrin clots, and that this is accompanied by a modified fibre architecture, rendering clots more resistant to lysis [21, 22]. Hess et al [20] expand on these previous findings by showing that clots from type 1 diabetic patients are more resistant to fibrinolysis, which appears to be due to altered clotting and lysis features of diabetic fibrinogen and C3. In a variety of experiments they demonstrate that clots generated from diabetic fibrinogen incorporate more C3, resulting in more consolidated clots. On the other hand, exposure of both diabetic and control fibrinogen to diabetic C3 produces clots more resistant to lysis. Furthermore, in diabetic individuals, clot lysis time was correlated with plasma C3 levels. The authors concluded that the effect of C3 on clot properties is more pronounced in diabetes. It was also suggested that systemic C3 levels and C3-mediated fibrin clot formation and lysis properties may be influenced via glycaemic control, although these were essentially uncontrolled observations that clearly need corroborating.

The mechanism of diabetes-dependent C3 incorporation into fibrin clots with concomitant effects on clot characteristics adds to our knowledge of the broad aspects of complement functioning in type 1 and, potentially also, type 2 diabetes, and may in due course be extended to related disorders, including obesity and NAFLD. Unfortunately, Hess et al [20] did not consider C3 activation in their experiments, which hampers full comprehension of the presented data. For a better understanding of the C3 coagulation/fibrinolysis interaction, it is essential to know to what extent C3 activation is involved. If the mechanism presented by Hess et al takes place basically without C3 activation, this would represent a completely novel, direct mode of C3 operating on CVD risk, whereby C3 would contribute to a hypofibrinolytic/prothrombotic state solely by its presence in clots, thereby modulating clot properties. In such a scenario, it will be essential to elucidate what intrinsic characteristics of diabetic C3 and fibrinogen underlie the observed changes in clot lysis properties. Glycation of C3 may be a plausible possibility [23]. It will also be interesting to see if similar observations with respect to the role of C3 in hypofibrinolysis can be made in type 2 diabetic individuals.

In summary, this commentary gives a brief overview of the emerging roles of C3, C3a and C3a-desarg/ASP in adipose tissue homeostasis, insulin resistance and (development of) diabetes and related cardiometabolic diseases. The epidemiological and experimental evidence presented supports a role for these complement factors in metabolic, atherosclerotic/atherothrombotic and microangiopathic processes. It should be noted that an increasing number of additional complement factors are reported to be expressed in adipose tissue in association with insulin resistance (for example, see [24]). Likewise, a range of additional complement components and regulators have also been shown to be involved in the process of atherosclerosis and atherothrombosis [25]. The current study by Hess et al [20] expands our knowledge of the multifaceted role of the complement system, C3 in particular, in the pathogenesis of diabetes-associated CVD. Further epidemiological, experimental and clinical studies are needed to differentiate between, and determine the relevance of, different aspects of complement function and activation in the complex and inter-related processes that are characteristic of cardiometabolic disorders.

## Abbreviations

ASP: Acylation-stimulating protein
C3aR: C3a receptor
C5L2: C5a-like receptor 2
CVD: Cardiovascular disease
MAC: Membrane-attack complex
NAFLD: Non-alcoholic fatty liver disease
TCC: Terminal complement complex
